# A comprehensive evaluation of the potential of three next-generation short-read-based plant pan-genome construction strategies for the identification of novel non-reference sequence

**DOI:** 10.3389/fpls.2024.1371222

**Published:** 2024-03-19

**Authors:** Meiye Jiang, Meili Chen, Jingyao Zeng, Zhenglin Du, Jingfa Xiao

**Affiliations:** ^1^ National Genomics Data Center, Beijing Institute of Genomics, Chinese Academy of Sciences and China National Center for Bioinformation, Beijing, China; ^2^ CAS Key Laboratory of Genome Sciences and Information, Beijing Institute of Genomics, Chinese Academy of Sciences and China National Center for Bioinformation, Beijing, China; ^3^ College of Life Sciences, University of Chinese Academy of Sciences, Beijing, China

**Keywords:** plant pan-genome, short-reads based construction strategies, evaluation, map-to-pan, iterative

## Abstract

Pan-genome studies are important for understanding plant evolution and guiding the breeding of crops by containing all genomic diversity of a certain species. Three short-read-based strategies for plant pan-genome construction include iterative individual, iteration pooling, and map-to-pan. Their performance is very different under various conditions, while comprehensive evaluations have yet to be conducted nowadays. Here, we evaluate the performance of these three pan-genome construction strategies for plants under different sequencing depths and sample sizes. Also, we indicate the influence of length and repeat content percentage of novel sequences on three pan-genome construction strategies. Besides, we compare the computational resource consumption among the three strategies. Our findings indicate that map-to-pan has the greatest recall but the lowest precision. In contrast, both two iterative strategies have superior precision but lower recall. Factors of sample numbers, novel sequence length, and the percentage of novel sequences’ repeat content adversely affect the performance of all three strategies. Increased sequencing depth improves map-to-pan’s performance, while not affecting the other two iterative strategies. For computational resource consumption, map-to-pan demands considerably more than the other two iterative strategies. Overall, the iterative strategy, especially the iterative pooling strategy, is optimal when the sequencing depth is less than 20X. Map-to-pan is preferable when the sequencing depth exceeds 20X despite its higher computational resource consumption.

## 1 Introduction

In 2005, Tettelin et al. introduced the pan-genome concept to encompass the entire gene set in *Streptococcus agalactiae* (Tettelin et al., 2005). Since then, this concept has gained widespread application in characterizing the collective genes of a species, encompassing core, dispensable, and private components. The advancement of sequencing technology, especially the prevalent next-generation short-read sequencing, has enabled large-scale pan-genome analysis in plants, extending beyond its initial application in microbes. By 2007, the pan-genome concept was introduced to maize (Morgante et al., 2007). After that, plenty of studies have delved into the plant pan-genomes of diverse species, such as poplar (Zhang et al., 2019), *Brachypodium distachyon* (Gordon et al., 2017), *Brassica oleracea* (Golicz et al., 2016), *Brassica napus* (Hurgobin et al., 2018), pepper (Ou et al., 2018), Medicago (Zhou et al., 2017), rice (Zhao et al., 2018), soybean (Li et al., 2014), hexaploid bread wheat (Montenegro et al., 2017), tomato (Gao et al., 2019), and sunflower (Hübner et al., 2019). These plant pan-genomics studies are pivotal in pinpointing key novel non-reference genes or sequences related to processes like signaling (Golicz et al., 2016), defense mechanisms (Gordon et al., 2017), resistance pathways (Bayer et al., 2019), important agricultural traits (Gao et al., 2019), and heterosis (Zhang et al., 2016).

Microbial pan-genome studies have benefited from well-established toolkits like Roary (Page et al., 2015), PGAP (Zhao et al., 2012), PanGP (Zhao et al., 2014), PanOCT (Fouts et al., 2012), and PANNOTATOR (Santos et al., 2013), while there is not a uniform strategy or pipeline for plant pan-genome construction. There are three plant pan-genome construction strategies based on next-generation sequencing short-reads. They can be summarized as the iterative individual (Golicz et al., 2016; Hurgobin et al., 2018; Hübner et al., 2019), the iterative pooling (Montenegro et al., 2017), and the map-to-pan (Hu et al., 2017; Sun et al., 2017; Zhou et al., 2017; Ou et al., 2018; Gao et al., 2019; Qin et al., 2021). All these three strategies construct a pan-genome based on a high-quality reference genome. For map-to-pan, the whole genome of each accession included in the pan-genome analysis is assembled and then aligned to the reference genome to obtain non-redundant novel sequences not existing in the reference genome. Unlike map-to-pan, unmapped or poorly mapped reads with reference genomes are first extracted. In the iterative pooling method, unmapped or poorly mapped reads from each accession are pooled and assembled in a metagenomic way. In the iterative individual approach, unmapped or poorly mapped reads are assembled directly for each accession, pooled, and removed redundancy. Two iterative strategies are used for pan-genome construction with large-scale samples due to their low requirement for low sequencing depth and computation resource consumption. In contrast, whole genome sequencing and assembly are needed in map-to-pan, so map-to-pan is suitable for pan-genome construction with a few samples. Some pan-genome studies have incorporated long reads from third-generation sequencing platforms, like in rice (Qin et al., 2021), soybean (Liu et al., 2020), sorghum (Tao et al., 2021), maize (Hufford et al., 2021), and *Raphanus sativus* (Zhang et al., 2021), while their widespread adoption is constrained by high sequencing expenses, especially in plant pan-genome projects with large-scale samples. Given the vast availability of published short-read sequencing data for numerous plant species, it is prevalent to construct plant pan-genomes based on next-generation short-reads.

Here, we thoroughly benchmark these three strategies for plant pan-genome construction, factoring in different sequencing depths and the number of samples included. We also compare the efficiency of these three strategies in recovering novel non-reference sequences with different lengths and repetitive content percentages. Additionally, we compare computational resource consumption among these three strategies, encompassing both time and memory. Our in-depth evaluation aims to shed light on the effectiveness of these three pan-genome construction strategies under varying conditions and guide researchers in choosing the optimal pan-genome construction strategy.

## 2 Materials and methods

### 2.1 Data sets

Our research collected 20 high-quality chromosome-level genome assemblies, gene annotation files, gene sequences, protein sequences, and PacBio long reads from the rice XI subtype (Qin et al., 2021) (**Supplementary Table 1**). We categorized these samples into five groups with 5, 8, 10, 15, and 20 samples, respectively. The group with 8 samples included all subtypes from XI-1B. It was used for benchmarking the influence of various sequencing depths, lengths, and repeat content percentages of novel sequences on these three strategies. The information from the other four groups was compared to examine how the sample number included affected the performance of these three strategies.

The ART-Illumina read simulation tool (Huang et al., 2012) was used to generate the simulated next-generation sequencing short-reads with depths of 5X, 10X, 20X, 30X, and 50X, with 20 high-quality chromosome-level genome assemblies as the reference. To evaluate the limitations of simulated reads, the real data of next-generation sequencing short-reads for the 9311 sample was downloaded from GSA (https://ngdc.cncb.ac.cn/gsa/) under Project ID PRJCA002103 and RunID CRR279354. These sequences were aligned to the reference genome using BWA-MEM (Li, 2013). MSU was used as a reference genome, and its genome sequence was downloaded from RiceRC (https://ricerc.sicau.edu.cn/RiceRC/download/downloadBefore). This genome assembly produced by the Rice Genome Annotation Project was initially located at the Institute for Genomic Research. It is now at Michigan State University (MSU) (Ouyang et al., 2007). Finally, sequencing depth, genome coverage, and other characteristics were calculated using the BAMDST toolkit (https://github.com/shiquan/bamdst). We generated the simulated sequencing data according to the average depth of real data for each chromosome. The characteristics of simulated data were calculated by the BAMDST toolkit and then compared with the characteristics of real data.

### 2.2 Construction of the testing data set

**Figure 1:**
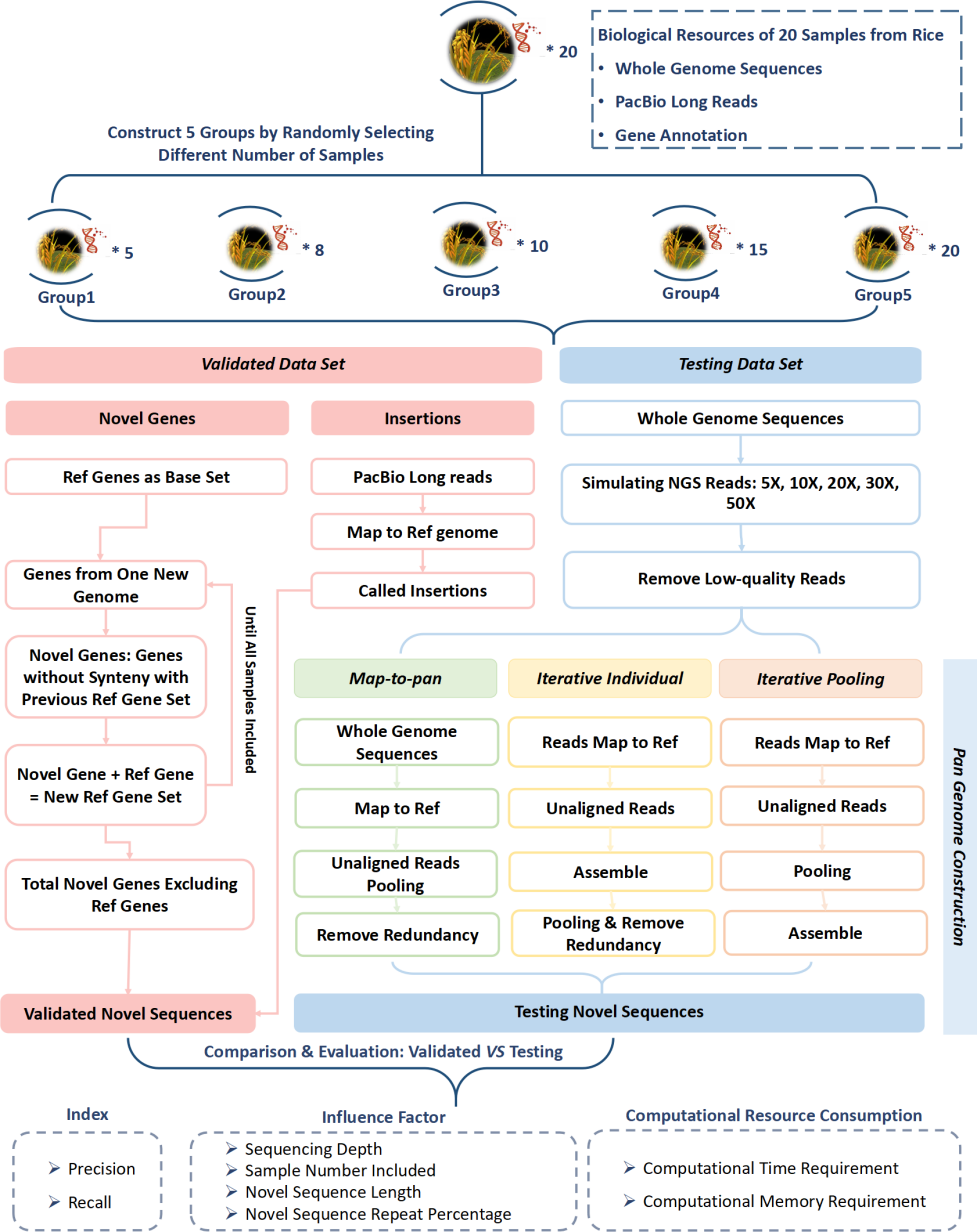
Workflow of evaluation for three plant pan-genome construction strategies based on next-generation short-reads.

Three pan-genome construction strategies, iterative individual, iterative pooling, and map-to-pan, utilized simulated short reads to create a test dataset for each group with different sample sizes (**Figure 1**). Each strategy underwent identical data pre-processing, which involved eliminating reads with over five Ns, trimming adapters, removing low-quality bases from the 5’ and 3’ ends when the quality score was consistently below 20, and discarding reads shorter than 30 bp. All pre-processing tasks were executed using a Perl script developed in-house, which was deposited in BioCode with ID BT007415 (https://ngdc.cncb.ac.cn/biocode/tools/BT007415).

For map-to-pan, high-quality reads were firstly collected for whole genome assembly using SOAPdenovo2 (Luo et al., 2012) through the *eupan assemble linearK* model in the EUPAN toolkit (Hu et al., 2017). The iterative k-mer was set to a range between 15 and 127 to optimize the assembly outcome. Secondly, the whole genome assembly of each sample was aligned to the reference genome via the MUMmer software (Kurtz et al., 2004). Those sequences not aligned with the reference genome with 90% identity and 90% coverage simultaneously were recognized as candidate novel non-reference sequences. Subsequently, each sample’s novel sequences were combined, and redundancy was eliminated using CD-HIT (Fu et al., 2012).

For the iterative individual, high-quality reads were initially mapped to the reference genome using BWA MEM (Li, 2013). Unmapped and poorly mapped reads and those with an edit distance of ≥ 8 were extracted for assembly by MEGAHIT (Li et al., 2015). Then, the contigs assembled from each sample were merged, and redundancy was removed with CD-HIT (Fu et al., 2012). For iterative pooling, high-quality reads were initially mapped to the reference genome using BWA-MEM (Li, 2013). Unmapped and poorly mapped reads with an edit distance of ≥ 8 were extracted and pooled. These pooling of unmapped or poorly mapped reads were assembled using MEGAHIT (Li et al., 2015).

For both iterative methods, the edit distance threshold was 8 to select poorly mapped reads. The length of almost all simulated reads was 83 bp, so if the edit distance was greater than 8, the mapping rate of a read to the reference genome was less than ~90%. They may be from highly diverse genomic regions of subspecies compared with the reference genome. So, these reads were also collected and combined with the unmapped reads for novel sequence assembly for two iterative methods.

Unlike the SOAPDENOVO2 for assembly in map-to-pan, we employed MEGAHIT to assemble those unmapped or poorly mapped reads in both iterative strategies to maximize the utilization of these reads. Since MEGAHIT was often utilized for microbial metagenome assembly, it performed better when reads exhibited greater heterogeneity, especially in iterative pooling, where unmapped or poorly mapped reads were pooled together for assembly.

### 2.3 Construction of the validated data set

The plant pan-genome consists of the gene-centric and sequence-centric pan-genome (Golicz et al., 2020). Here, novel genes identified from gene-based pan-genome and insertions identified from sequence-based pan-genome were combined as the validated data set.

For gene-centric pan-genome construction, there were two kinds of strategies including synteny-based, such as in rice (Qin et al., 2021), and gene clustering-based, such as in *Brachypodium distachyon* (Gordon et al., 2017) using GET_HOMOLOG-EST (Contreras-Moreira et al., 2017), soybean (Liu et al., 2020) using OrthoMCL (Li et al., 2003), rice (Shang et al., 2022) using OrthoFinder (Emms and Kelly, 2019). Besides, GENESPACE can cluster genes across multiple genomes (Lovell et al., 2022). Here, we used a synteny-based method. Protein sequences related to the longest gene transcript and information on the gene location for each of the 20 samples from Qin et al (Qin et al., 2021) were used for the gene-based pan-genome construction for each of the 5 groups. All genes of the nuclear genome’s 12 chromosomes from MSU (V.7.0 http://rice.plantbiology.msu.edu) were used as the base. Genes from a new genome were aligned against a reference gene set using BLASTP software (Altschul et al., 1990) and gene synteny was analyzed using MCSCANX software (Wang et al., 2012). Those genes that did not show synteny with the reference gene set were considered novel genes. These novel genes were then added to the former reference gene set to form a new reference gene set. These steps were repeated until all samples were included. The reference gene set and identified novel genes from the final step were combined as the pan-gene set. Novel genes from each step were combined and then aligned to the MSU reference genome using MUMmer (Kurtz et al., 2004). Genes with high similarity (identity ≥ 90% and coverage ≥ 90%) with the MSU reference genome were discarded to exclude the false positives. The remaining gene set was used for further analysis.

To compare the consistency of the gene-based pan-genome from the synteny-based method and gene-clustering-based methods, OrthoFinder was used to construct the gene-based pan-genome with the reference genome and extra 5, 8, 10, 15, and 20 samples. Those gene groups not containing genes from MSU were considered novel gene groups that did not exist in the reference genome.

Sequence-based pan-genome was constructed as complementary to gene-based pan-genome. Here, insertions compared with the reference genome from each sample for each of the 5 groups were considered novel sequences absent from the reference genome. PacBio long reads of each sample were first mapped to the MSU reference genome by pbmm2 software (https://github.com/PacificBiosciences/pbmm2) with default parameters. After this, structural variations were called and genotyped using pbsv software (https://github.com/PacificBiosciences/pbsv) using default parameters. Entries related to insertions were extracted. Then, these insertions were merged at the group level using SURVIVOR software (Jeffares et al., 2017). Those insertions ≤ 50 bp in length or had supporting reads of ≤ 20 were excluded. To eliminate the false positive introduced during insertion identification, the remaining insertion sequences were then aligned to the genome of each sample in each of the 5 groups. Those insertions not having a high similarity (identity ≥ 90% and coverage ≥ 90%) with the genome sequences were excluded.

The RepeatMasker tool (Chen, 2004) was employed for the validated data set to detect repetitive elements, using rice as the model species.

### 2.4 Recall and precision definition

The sequences from the testing data set were aligned to sequences from the validated data set using the MUMmer software (Kurtz et al., 2004). When different sequences from the testing data sets were aligned to the same sequences from the validated data set, and they had an overlap of 90% or more, these sequences from the testing data sets and their recovered regions for sequences from the validated data set were combined. For each sequence from the validated data set, its coverage was defined as the ratio of recovered length by sequences from the testing data set to its whole length. If the coverage was ≥ 0.5, this sequence from the validated data set was considered a recovered sequence. The recall value was defined as the ratio of the number of recovered ones to the total number of sequences from the validated data set.

For each of the 5 groups, sequences from the testing data set were aligned to all genomes in that group. Those sequences with a high similarity (90% identity and 90% coverage) were considered as precise sequences. The precision value was defined as the ratio of the number of precise ones to the total number of sequences from the testing data set.

## 3 Results

### 3.1 The characteristics of the testing and validated data set

**Figure 2:**
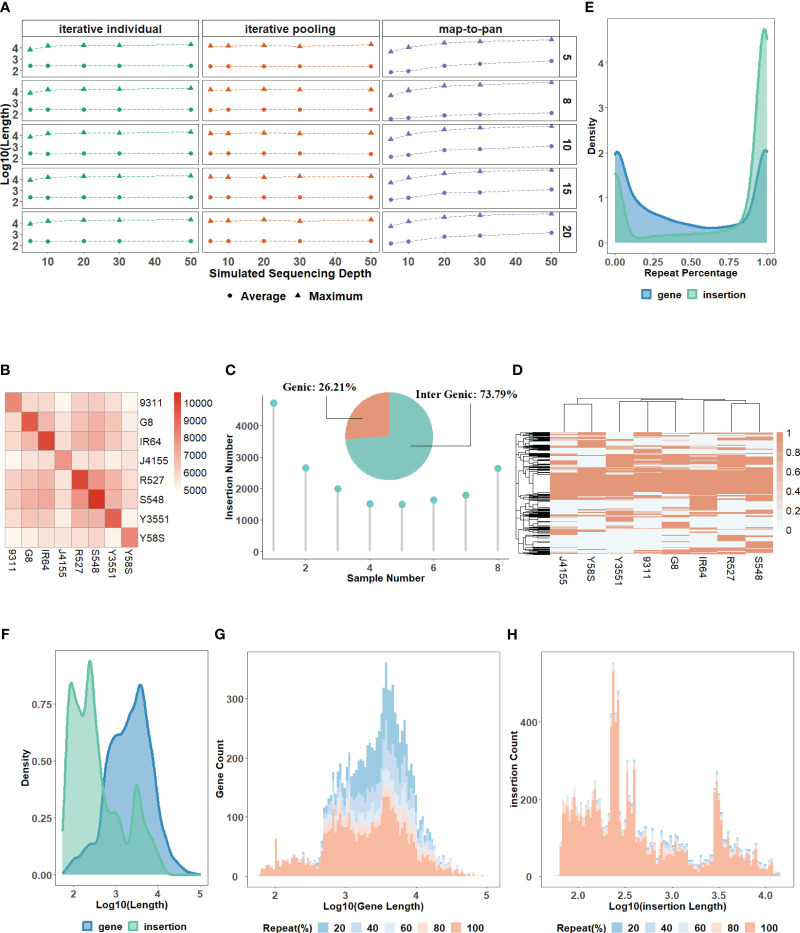
**(A)** The average and maximum lengths of assembled contigs for three strategies across varied sequencing depths. **(B)** A heatmap of the overlapping number of insertions between paired samples in the group consisting of 8 samples. **(C)** A pie chart showing the percentage of insertions found in genic versus intergenic regions and the distribution of insertion numbers as samples increase in the group consisting of 8 samples. **(D)** A heatmap of the presence and absence profile for insertions across samples in the group consisting of 8 samples. The distribution for the repeat content percentage **(E)** and length **(F)** of novel sequences from the validated data set for the group consisting of 8 samples. The distribution for the count of novel genes **(G)** and insertions **(H)** with different lengths and repeat content percentages in the group consisting of 8 samples.

The characteristics of the testing data set. All the simulated next-generation short-reads with sequencing depths of 5X, 10X, 20X, 30X, and 50X for 20 samples have a high-quality read rate of ≥99% (**Supplementary Table 2**). By comparing the characteristics between simulated and real data, we find that the simulated reads have almost identical or even higher genome coverage than the real data under the same sequencing depth (**Supplementary Table 3**). This indicates the availability of simulated data for evaluation. However, there are some biases in simulated data. For example, the rate of singletons and reads pairs mapping to different chromosomes of simulated data is lower than in real data (**Supplementary Table 4**). These simulated reads after preprocessing are used to construct the testing data set using three strategies for each of the 5 groups (**Supplementary Table 5**). For map-to-pan, optimal k-mers used for whole genome assembly for different samples are different, highlighting the necessity for an iterative k-mer strategy (**Supplementary Figure 1**). When sequencing depth increases, the length of assembled contigs of map-to-pan increases, while sequencing depth has no significant influence on both iterative methods (**Figure 2A**).

The characteristics of the validated data set. For gene-based pan-genome, the ratio of core genes decreases with sample size increases, and this ratio stabilizes around 50% when the sample size reaches 6 or more (**Supplementary Table 6**). For the group with MSU and the other 8 samples, synteny-based methods can find 18,500 (91.67%) of 20,179 gene groups from OrthoFinder. After filtering, all 13,078 novel genes identified from the synteny-based method are included in the results from the OrthoFinder. This further demonstrated the usability of synteny-based methods in novel gene identification. For sequence-based pan-genome by 8 samples, the insertion counts diverge among samples, and their overlaps with each other are not uniform (**Figure 2B**). Insertions are predominantly localized in intergenic regions, indicating that insertions can be used as a complement to novel genes (**Figure 2C**). The insertions have different distribution patterns among different samples, further supported by the insertion presence and absence profile (**Figure 2D**). The characteristics of sequence-based pan-genome are consistently observed in the other 4 groups (**Supplementary Figure 2**). The summary of novel genes and insertions for each of the 5 groups is shown in **Table 1**. Insertions have a higher repeat percentage than the novel genes (**Figure 2E**), retroelements and DNA transposons emerge as the predominant repeat elements in them (**Supplementary Table 7**). However, their overall lengths are less than the novel genes (**Figure 2F**). The repeat percentage of novel genes is the highest at the longest and shortest ones (**Figure 2G**), while for insertions, they consistently show a high repeat percentage for all lengths (**Figure 2H**).

**Table 1:** Statistics of novel genes and insertions from the validated data set for each of the 5 groups.

Type	Sample Number	# Seqs	Total Size (bp)	Mean Length (bp)	Repeat Percentage
Novel Genes	5	9,697	39,114,313	4033.70	46.02%
	8	13,078	51,527,357	3940.00	46.19%
	10	15,306	59,557,869	3891.10	46.30%
	15	19,901	79,273,953	3983.40	46.38%
	20	24,792	98,210,643	3961.40	46.38%
Insertions	5	13,082	12,528,436	957.70	44.44%
	8	15,047	16,504,941	1096.90	44.77%
	10	17,109	20,891,729	1221.10	45.11%
	15	18,756	25,039,572	1335.00	45.24%
	20	19,959	27,876,840	1396.70	45.37%

### 3.2 Evaluation of the influence of sequencing depth on three pan-genome construction strategies

**Figure 3:**
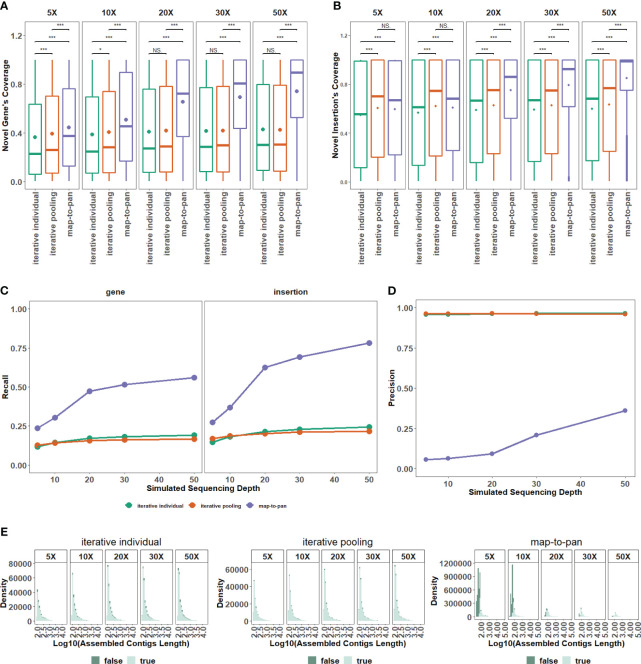
The impact of sequencing depth on three strategies. **(A)** The distribution for recovered coverage of sequences from the testing dataset to novel genes from the validated dataset for the three strategies across varied sequencing depths. **(B)** The distribution for recovered coverage of sequences from the testing dataset to insertions from the validated dataset for the three strategies across varied sequencing depths. **(C)** Recall distribution for the three strategies across various sequencing depths. **(D)** Precision distribution for the three strategies across various sequencing depths. **(E)** Distribution of assembled length, categorized by false and true tags, for the three pan-genome construction strategies of the plant. NS means P > 0.05, * means P ≤ 0.05, ** means P < 0.01, *** means P < 0.001.

Testing and validated data sets from the group with 8 samples are utilized to evaluate the different efficiency of three pan-genome construction strategies under different sequencing depths. For the coverage of novel genes from the validated data set under all different sequencing depths (**Figure 3A**) and insertions from the validated data set under 20X or more sequencing depth (**Figure 3B**), the difference is significant between map-to-pan and the other two iterative strategies, highlighting the different performance of map-to-pan and the other two iterative strategies. The difference is significant between iterative individual and iterative pooling for the coverage of novel genes under 10X or less sequencing depth (**Figure 3A**) and insertions (**Figure 3B**) under all different sequencing depths. Iterative pooling has a slightly higher average coverage for novel sequences from the validated data set than iterative individual, especially when sequencing depth is 10X or less. The main reason is that iterative pooling gathered all unmapped or poorly mapped reads for assembly, comparable to increasing the sequencing depth.

Map-to-pan has the highest recall value, and the other two iterative strategies have nearly identical lower recall values (**Figure 3C**). Specifically, the recall value of both novel genes and insertions from the validated data set is lower than 0.25 for two iterative strategies under all sequencing depths. For map-to-pan, the recall value of novel genes from the validated data set is around 0.5, and of insertions from the validated data set is around 0.75 under 50X sequencing depth.

Conversely, map-to-pan has the lowest precision value, and the other two iterative strategies have almost identical precision values (**Figure 3D**). Those sequences that are not precisive, are mainly from short sequences for map-to-pan and have a consistent distribution across all lengths for the other two iterative strategies (**Figure 3E**).

Overall, higher sequencing depths improve map-to-pan performance, including its coverage and recall for novel sequences from the validated data set (**Figure 3A–C**), and precision (**Figure 3D**). However, there needs to be obvious evidence to support the influence of sequencing depth on the other two iterative strategies.

### 3.3 Impact of sample size on three pan-genome construction strategies

In pan-genome research, including more samples will introduce more genomic diversity and biological information unless the current pan-genome of certain species is closed. A closed pan-genome means adding new genomes or samples will not induce the increase in pan-genome size, which depends on the frequency of gene exchange between subspecies and whether enough samples are included. Therefore, the number of samples included is vital in pan-genome construction.

**Figure 4:**
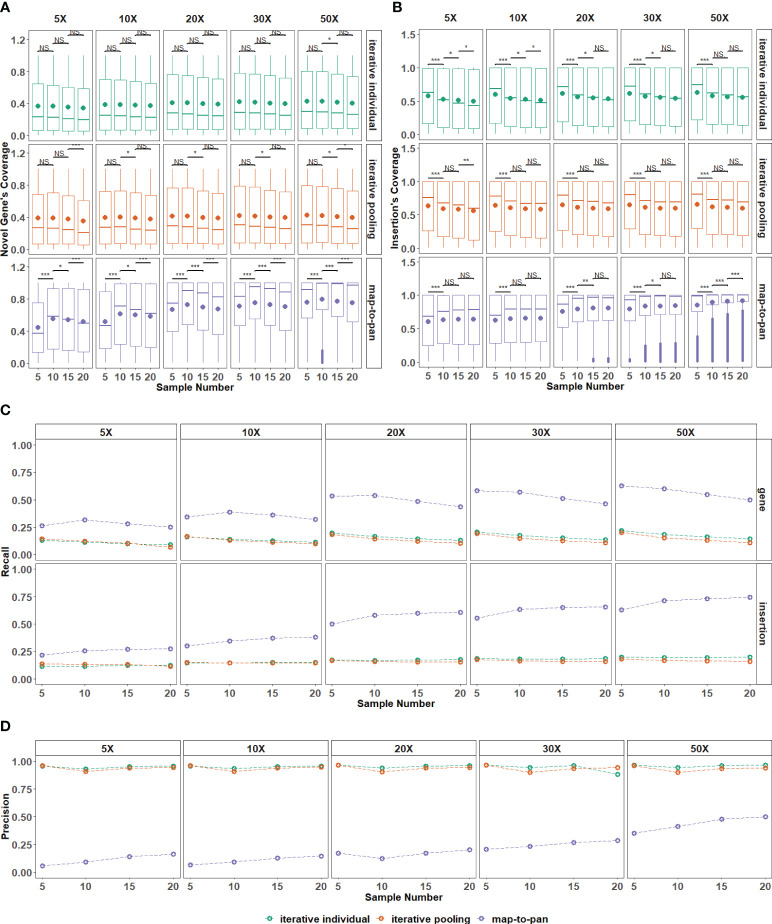
The impact of the number of samples included on three strategies. **(A)** The distribution for recovered coverage of sequences from the testing dataset to novel genes from the validated dataset for the three strategies across various sample numbers included. **(B)** The distribution for recovered coverage of sequences from the testing dataset to insertions from the validated dataset for the three strategies across various sample numbers included. **(C)** Recall distribution for the three strategies across various sample numbers included. **(D)** Precision distribution for the three strategies across various sample numbers included. NS means P > 0.05, * means P ≤ 0.05, ** means P 0.01, *** means P < 0.001.

For sequences from the map-to-pan strategy, the difference in their coverage for novel genes from the validated data set is significant among different sample sizes with all sequencing depths. At the same time, there is no significance for both iterative strategies (**Figure 4A**). Conversely, for sequences from these three strategies, their coverage for insertions from the validated data set is similar among different sample sizes, except for the map-to-pan strategy under 50X sequencing depth (**Figure 4B**).

Recall and precision values are further used to evaluate sample size influence on these three strategies. For map-to-pan, their recall value for novel genes decreases as sample size increases, while for insertions, their recall value increases as sample size increases (**Figure 4C**). For two iterative strategies, the sample size does not significantly influence their recall value for both novel genes and insertions from the validated data set. There is no obvious difference between iterative individual and iterative pooling.

Higher sequencing depth can improve the coverage and recall for novel sequences from the validated data set of map-to-pan with an expanded sample size but does not affect both iterative strategies. This indicates the limited capability of iterative strategies for novel sequence identification, no matter the sample size or sequencing depth. Map-to-pan has the lowest precision value under different sample sizes, while there is a positive correlation between precision value and sample size, such a relationship is not observed for the two iterative methods (**Figure 4D**).

### 3.4 Comparison of three pan-genome construction methods’ performance with the different novel sequence length

Novel sequences from the validated data set are divided into four length-based categories: SS, S, M, and L for both novel genes and insertions (**Supplementary Table 8**). SS-tagged novel sequences have lengths from 50 bp to 100 bp, S-tagged novel sequences have lengths from 100 bp to 1000 bp, M-tagged novel sequences have lengths from 1000 bp to 10000 bp, L-tagged novel sequences have lengths larger than 10000 bp. Most novel genes fall in the M category, whereas most insertions are in the S category.

For sequences from all three strategies, there is a negative relationship between their coverage for novel sequences from the validated data set and the length of the novel sequences from the validated data set for both novel genes and insertions (**Supplementary Figure 3A, B**). Increased sequencing depth improves the recovered coverage of sequences from map-to-pan for novel sequences from the validated data set (**Supplementary Figure 3A, B**) and the length of recovered novel sequences from the validated data set, especially for insertions (**Supplementary Figure 3C**). The overall recall value is lower for the SS and L categories than the S and M categories for all three strategies (**Supplementary Figure 3D**). The recall value drops as the length of novel sequences from the validated data set increases for two iterative strategies under all sequencing depths and for map-to-pan under 10X or less sequencing depth. Increased sequencing depth improves the map-to-pan’s recall for novel sequences with different lengths but has no significant effect on the two iterative methods.

Regarding recall value, the map-to-pan strategy outperforms the other two iterative strategies for different length categories except for L. Additionally, no significant difference exists between the individual and pooling iterative strategy across all length categories.

### 3.5 Diverse efficiency of three pan-genome construction methods in response to novel sequences’ repeat content percentage

Novel genes and insertions from the validated data set are divided into ten groups based on their repeat content percentage, using intervals of 0.10. The majority of these genes and insertions are found within the [0, 0.1] and (0.9, 1] intervals (**Supplementary Table 9**).

For sequences from all three pan-genome construction strategies, their recovered coverage of novel sequences from the validated data set decreases as the repeat content percentage increases (**Supplementary Figure 4A, 4B**). Novel sequences with repeat percentages in the ranges of [0, 0.25] and [0.75, 1] are more easily identified by these three methods (**Supplementary Figure 4C**).

The recall value is negatively associated with the repeat content percentage for the two iterative strategies under all sequencing depths and for the map-to-pan technique under 10X or less sequencing depth (**Supplementary Figure 4D**). Sequencing depth can improve the recall value of map-to-pan for novel sequences with different repeat content percentages but has no significant effect on the two iterative methods. Overall, the map-to-pan strategy has a higher recall value than the other two iterative strategies, especially for those novel sequences with higher repeat percentages. The distinction between the iterative individual and iterative pooling strategies is subtle under different repeat content percentages.

### 3.6 Time and memory consumption comparison among three pan-genome construction methods

The map-to-pan strategy demands considerably greater computational resources regarding memory and time than the other two iterative methods (**Table 2**). The main computational burden for the map-to-pan strategy arises from assembling the whole genome for every sample included. At a sequencing depth of 30X, it uses about 62GB of memory and takes approximately 212 minutes for each sample, utilizing 4 CPUs. Assembling unmapped or poorly mapped reads for the iterative individual strategy uses only around 10MB and takes about 18 minutes per sample. For the iterative pooling strategy, assembling pooled unmapped or poorly mapped reads consumes nearly 10MB of memory and takes about 115 minutes to construct a pan-genome with 8 samples, operating on 4 CPUs. The second highest computational demand for the map-to-pan strategy comes from aligning the assembled genome of each sample to the reference genome. In the case of the two iterative methods, only the assembly of unmapped or poorly mapped reads is aligned to the reference genome, thus requiring significantly less memory and time than map-to-pan.

**Table 2:** Memory and time requirements for three pan-genome construction strategies at the sequencing depth of 30X for the 9311 sample.

Steps	Map-to-pan			Iterative Individual			Iterative Pooling		
	Mem	Time	CPU	Mem	Time	CPU	Mem	Time	CPU
Filter low-quality reads	1.3M/sample	~47mins/sample	1/sample	1.3M/sample	~47mins/sample	1/sample	1.3M/sample	~47mins/sample	1/sample
Map to reference genome and extract unmapped reads	…	…	…	5.4G/sample	~208mins/sample	4/sample	5.4G/sample	~208mins/sample	4/sample
MEGAHIT assembles individual unmapped reads	…	…	…	10M/sample	~18mins/sample	4/sample	…	…	…
Individual unmapped reads pooling and assemble for 8 samples	…	…	…	…	…	…	10M/sample	~115mins/sample	4/sample
Pool assembled contigs from individual unmapped reads and remove redundancy	…	…	…	350M/sample	~2mins/sample	4/sample	…	…	…
Whole genome assembly	~63G/sample	~212mins/sample	4/sample	…	…	…	…	…	…
Map whole genome assembly to reference	~480M/sample	~29mins/sample	4/sample	…	…	…	…	…	…
Extract unaligned contigs	–	~1min/sample	1/sample	…	…	…	…	…	…
Pool unaligned contigs and remove redundancy	~860M/sample	~13mins/sample	4/sample	…	…	…	…	…	…
									
In total	63G/sample	~5hrs/sample	4/sample	5.4G/sample	~4.5hrs/sample	4/sample	5.4G/sample	~4hrs/sample	4/sample

The computational resources are evaluated based on 9311 samples with 30X sequencing depth if a single sample is considered. If population statistics are needed, 8 samples, including 9311, G8, IR64, J4155, R527, S548, Y3551, and Y58S, are evaluated. All information is just based on 30X sequencing depth; if more sequencing depth and more samples are analyzed, then the time and memory will increase correspondingly. At 20X sequencing depth, for whole genome assembly mapping to reference, time and memory are also larger than that with 30X sequencing depth due to its large assembled genome size with a high false positive rate.

For both two iterative methods, the most resource-intensive step is the alignment of whole-genome sequencing reads from each sample included in the pan-genome construction to the reference genome. This step requires about 5.4GB of memory and an estimated 202 minutes per sample when using 4 CPUs for each sample.

## 4 Discussion

The pan-genome study proves effective for plant genomic studies because it aims to encompass all genomic diversity of a certain species, which is important for the deep understanding of evolution and providing more novel genomic targets for breeding. It aids in identifying crucial novel non-reference genes or sequences associated with signaling (Golicz et al., 2016), defense mechanisms (Gordon et al., 2017), resistance pathways (Bayer et al., 2019), vital agricultural attributes (Gao et al., 2019), and heterosis (Zhang et al., 2016). Currently, three strategies based on next-generation sequencing short-reads are utilized for constructing the plant pan-genome, they can be summarized as iterative individual, iterative pooling, and map-to-pan. They have different performances under different conditions. This diversity complicates the integration or comparison of pan-genome information for the same species from different projects and makes it difficult for users to select the optimal pan-genome construction strategy. Hence, we performed the first comprehensive evaluation of these three strategies considering the sequencing depths, sample sizes, length and repeat content percentage of novel sequence, and computational resource consumption.

Our findings indicate that: (1) map-to-pan has the highest recall but lowest precision value, whereas the two iterative strategies have lower recall but higher precision values; (2) the number of samples, the length of novel sequences, and the percentage of repeat content are inversely related to the recall value of these three pan-genome construction strategies, primarily because an increased number of samples brings more complexity, and new sequences with larger length and a higher percentage of repeat content are challenging to be assembled just based on next-generation short-reads; (3) higher sequencing depth can enhance the performance of map-to-pan, but it doesn’t affect the other two iterative strategies; (4) regarding the consumption of computational resources, map-to-pan requires significantly more than the other two iterative strategies, particularly at higher sequencing depths. Generally, the iterative method, particularly the iterative pooling method, is optimal when the sequencing depth is lower than 20X, considering recall and precision value. However, map-to-pan performs better with sequencing depths greater than 20X, even though it demands more computational memory and time.

However, there are some limitations in our evaluation. First, we only included a single species (rice) in our assessment. These three short-reads-based strategies for plant pan-genome construction may perform better in species with simpler genomes, such as *Arabidopsis thaliana*, and worse in species with more complex genomes, such as barley. Secondly, certain assembly and mapping software are used for these three strategies in our evaluation, while the choice of different software may also impact the evaluation results. Thirdly, we only used a synteny-based method for gene-based pan-genome construction. The core gene ratio differs slightly between these two methods of OrthoFinder and synteny-based. Fourthly, the choice of assessment data also influences the evaluation results. Here, we selected simulated data for evaluation, which needs to fully characterize the real data results. Meanwhile, we evaluated the performance of pan-genome construction strategies based on short reads. Still, it would be better to construct the pan-genome by a combination of short and long reads, such as in rice (Qin et al., 2021), soybean (Liu et al., 2020), sorghum (Tao et al., 2021), maize (Hufford et al., 2021), and *Raphanus sativus* (Zhang et al., 2021).

## Data availability statement

Publicly avaliable datasets were analyzed in this study. This data can be found here: Whole genome sequences, gene annotation files, gene sequences, and protein sequences of 20 rice samples are from Qin et al. (Qin et al., 2021). They can be downloaded from the RiceRC database via https://ricerc.sicau.edu.cn/. The PacBio long reads and real next-generation short reads of the 9311 sample are obtained from GSA under Project ID (PRJCA002103) via https://ngdc.cncb.ac.cn/gsa/. The Perl script used for data preprocessing is available via https://ngdc.cncb.ac.cn/biocode/tools/BT007415.

## Author contributions

MJ: Conceptualization, Data curation, Formal analysis, Investigation, Methodology, Resources, Software, Visualization, Writing – original draft, Writing – review & editing. MC: Writing – review & editing. JZ: Writing – review & editing. ZD: Supervision, Writing – review & editing. JX: Project administration, Supervision, Writing – review & editing.

## Supplementary material

Supplementary Figure 1 The optimal k-mer used in the whole genome assembly for each of the 20 rice samples using *eupan assemble linearK* model from the EUPAN toolkit.

Supplementary Figure 2 The heatmap of the overlapping number of insertions between paired samples in groups consisting of 5 **(A)**, 10 **(B)**, 15 **(C)**, and 20 **(D)** samples. The heatmap of the presence and absence profile of insertions across samples in the group consisting of 5 **(E)**, 10 **(F)**, 15 **(G)**, and 20 **(H)** samples. The distribution of insertion numbers as samples increase in the group consisting of 5 **(I)**, 10 **(J)**, 15 **(K)**, and 20 **(L)** samples. The pie chart shows the percentage of insertions found in genic versus intergenic regions in the group consisting of 5 **(M)**, 10 **(N)**, 15 **(O)**, and 20 **(P)** samples.

Supplementary Figure 3 The effect of the length for novel sequences on three strategies. **(A)** The distribution for recovered coverage of sequences from the testing dataset to novel genes from the validated dataset for the three strategies across various lengths. **(B)** The distribution for recovered coverage of sequences from the testing dataset to insertions from the validated dataset for the three strategies across various lengths. **(C)** Density of recovered length of sequences from the testing dataset to novel genes and insertions from the validated dataset for the three strategies across various lengths of novel sequences from the validated date set. **(D)** Recall distribution for the three strategies across various lengths of novel sequences from the validated data set. Length categories are defined as SS: 50-100bp; S: 100-1,000bp; M: 1,000-100,000bp; L: >100,000bp.

Supplementary Figure 4 The effect of repeat content percentage for novel sequences on three strategies. **(A)** The distribution for recovered coverage of sequences from the testing dataset to novel genes from the validated dataset for the three strategies across various repeat content percentages of novel sequences from the validated dataset. **(B)** The distribution for recovered coverage of sequences from the testing dataset to insertions from the validated dataset for the three strategies across various repeat content percentages of novel sequences from the validated dataset. **(C)** Density of recovered repeat content percentages across the three pan-genome construction strategies. **(D)** Recall distribution for the three strategies across various repeat content percentages of novel sequences from the validated data set. 0 means novel sequences from the validated data set have repeat content percentage [0,0.1], 1 means novel sequences from the validated data set have repeat content percentage (0.1,0.2], 2 means novel sequences from the validated data set have repeat content percentage [0.2,0.3], 3 means novel sequences from the validated data set have repeat content percentage [0.3,0.4], 4 means novel sequences from the validated data set have repeat content percentage [0.4,0.5], 5 means novel sequences from the validated data set have repeat content percentage [0.5,0.6], 6 means novel sequences from the validated data set have repeat content percentage [0.6,0.7], 7 means novel sequences from the validated data set have repeat content percentage [0.7,0.8], 8 means novel sequences from the validated data set have repeat content percentage [0.8,0.9], 9 means novel sequences from the validated data set have repeat content percentage (0.9,1].
