# Supplementary figures and images for: A comprehensive evaluation of the potential of three next-generation short-read-based plant pan-genome construction strategies for the identification of novel non-reference sequence
Source: Front Plant Sci. 2024 Mar 19;15:1371222. doi: 10.3389/fpls.2024.1371222 (PMC10986365; doi:10.3389/fpls.2024.1371222)

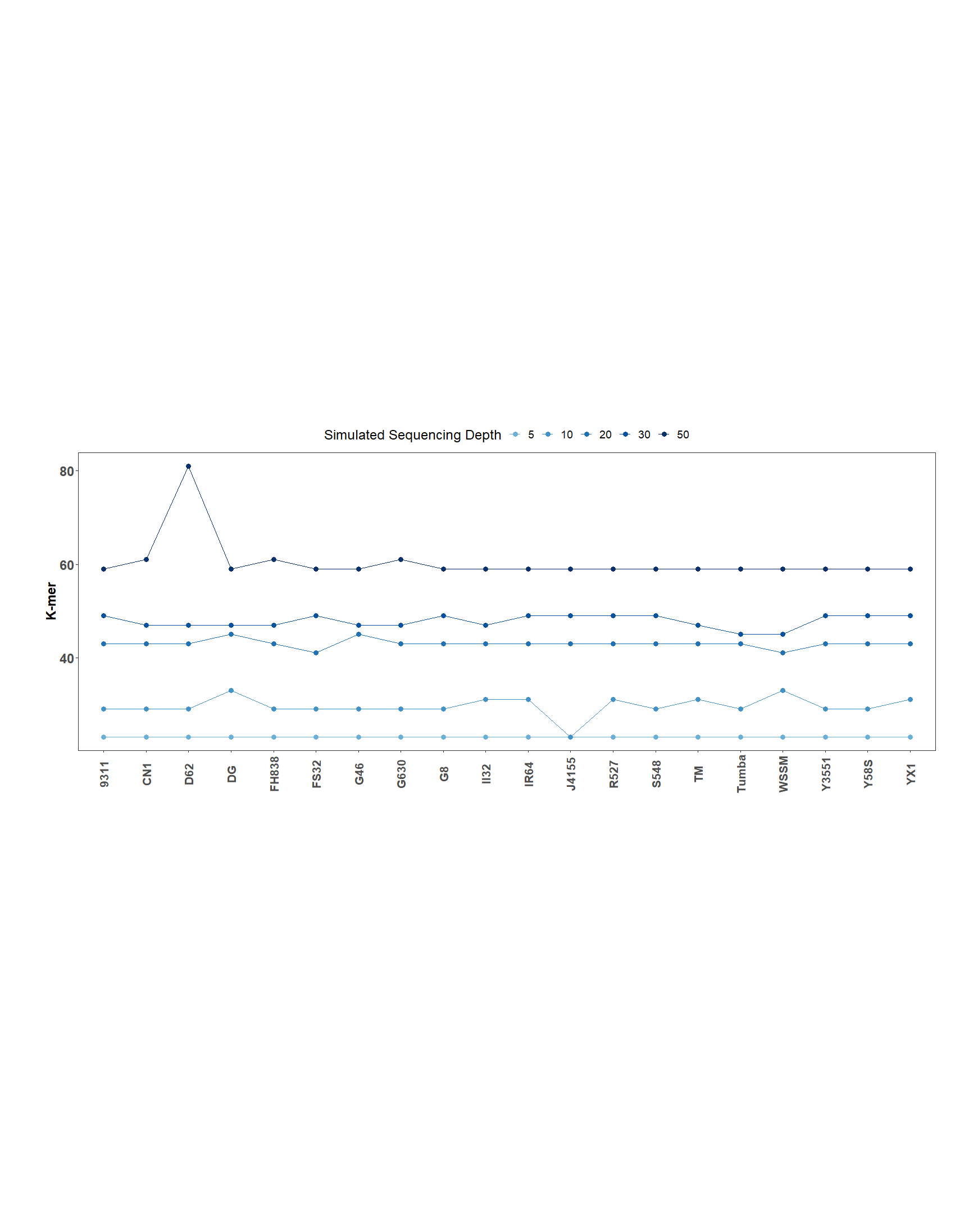

Supplement: Supplementary Figure 1 — The optimal k-mer used in the whole genome assembly for each of the 20 rice samples using eupan assemble linearK model from the EUPAN toolkit. [file DataSheet_1.zip › Data Sheet 1/Supplementary Figure 1.tif]

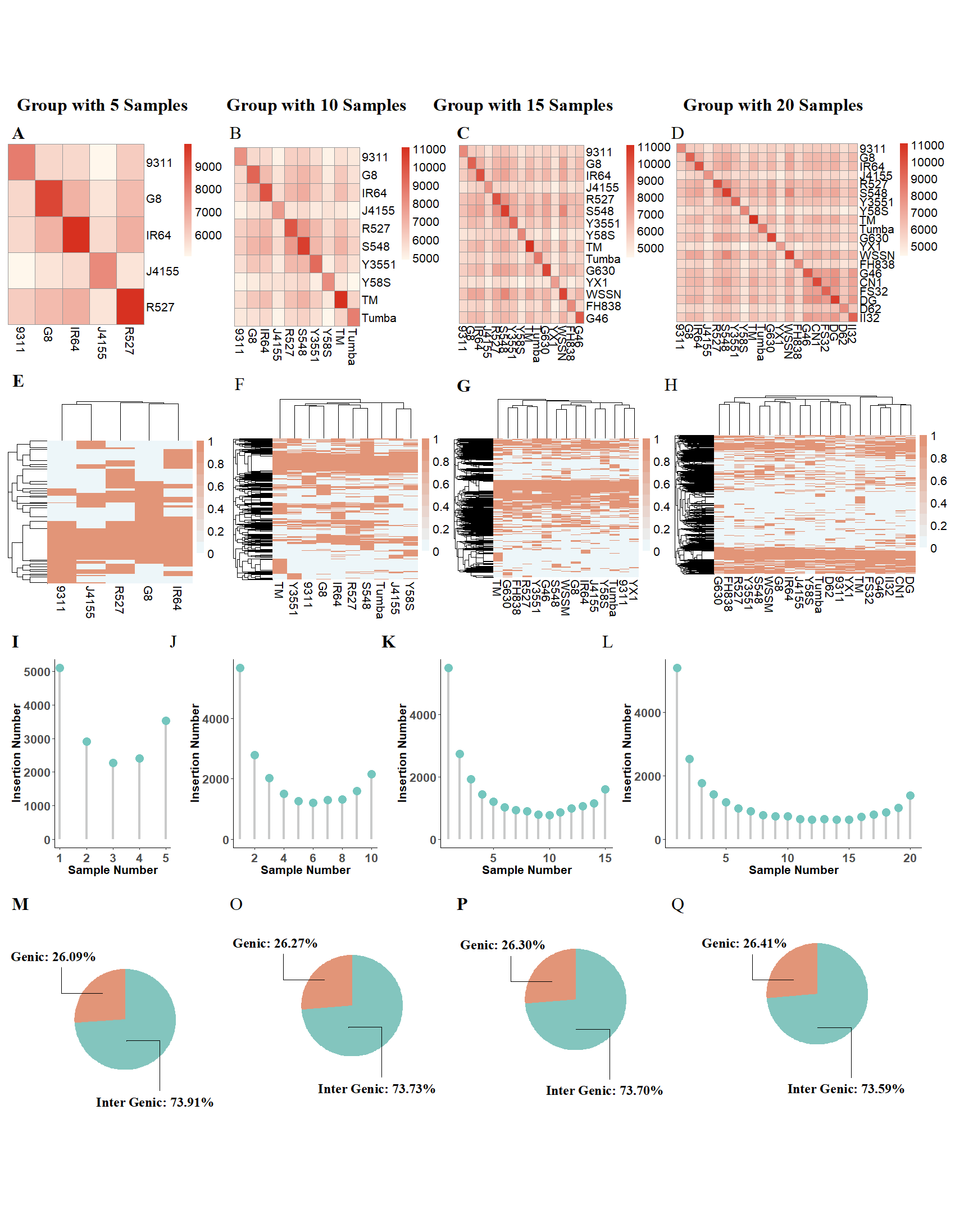

Supplement: Supplementary Figure 1 — The optimal k-mer used in the whole genome assembly for each of the 20 rice samples using eupan assemble linearK model from the EUPAN toolkit. [file DataSheet_1.zip › Data Sheet 1/Supplementary Figure 2.tif]

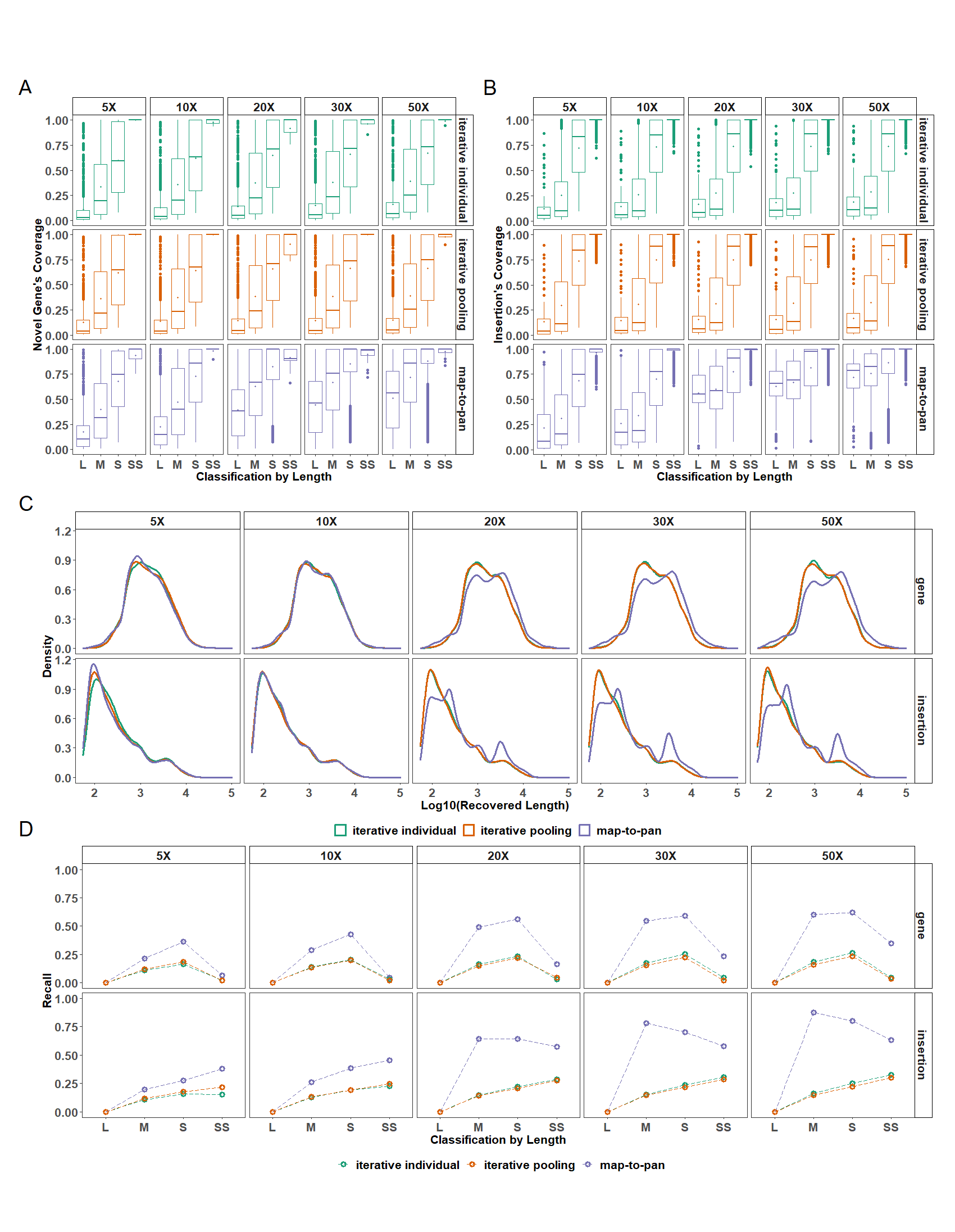

Supplement: Supplementary Figure 1 — The optimal k-mer used in the whole genome assembly for each of the 20 rice samples using eupan assemble linearK model from the EUPAN toolkit. [file DataSheet_1.zip › Data Sheet 1/Supplementary Figure 3.tif]

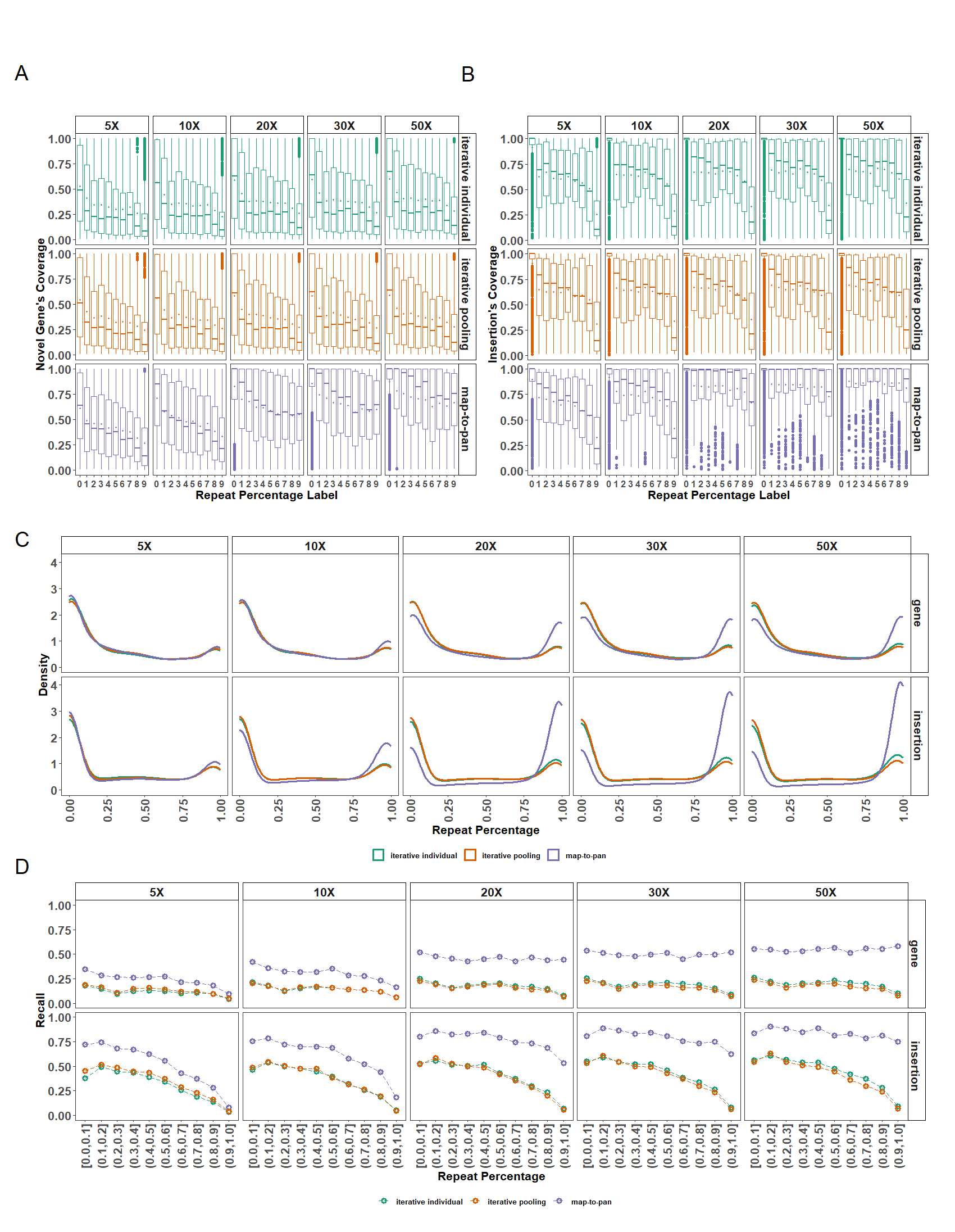

Supplement: Supplementary Figure 1 — The optimal k-mer used in the whole genome assembly for each of the 20 rice samples using eupan assemble linearK model from the EUPAN toolkit. [file DataSheet_1.zip › Data Sheet 1/Supplementary Figure 4.tif]
